# Airway function in adults with mild-to-moderate scoliosis treated in adolescence with specific physical exercises. An ongoing, case-control study

**DOI:** 10.1186/1748-7161-7-S1-O55

**Published:** 2012-01-27

**Authors:** M Plaszewski, R Nowobilski, P Kowalski, J Terech, M Cieslinski, I Cieslinski

**Affiliations:** 1 University School of Physical Education, Warsaw, Poland, Faculty of Physical Education in Biala Podlaska, Poland; 2 Department of Medicine, Jagiellonian University School of Medicine, Cracow, Poland; 3 Higher School of Administration, Bielsko-Biala, Poland; 4 Complex Hospital for Tuberculosis and Pulmonary Diseases, Bystra Slaska, Poland

## Background

Scoliosis can lead to a decrease in total lung capacity (TLC) and alterations in maximal flow – volume curves, associated with structural deformities and curve magnitude, but also with chronicity of the problem and respiratory muscle inefficiency [1-4]. However, evidence confirming the assumption of beneficial, long lasting influence of scoliosis specific exercise on respiratory function is lacking.

## Purpose

We aimed to analyze respiratory function in adults with history of participation in a scoliosis – specific exercise program, in comparison to normative values and to age-matched subjects, with reference to confounders: smoking and physical activity.

## Materials and methods

Maximal flow-volume curves, ventilatory parameters (vital capacity - VC, forced VC in exertion and in insertion = FVCin and FVCex) and TLC values were analyzed in 25 adults (22 females), who attended in adolescence (from 1984 to 1995, at initial age of 11 - 13) the Centre of Corrective and Compensatory Gymnastics, Bielsko–Biala, Poland. The WHO General Physical Activity Questionnaire was also completed. The non-parametric rang Kruskal-Wallis ANOVA was performed among subgroups with moderate and mild scoliosis (>40° Cobb, n=3; 25-39°, n=2; 10-24°, n=20, respectively) and compared to 17 age–matched normal controls (11 females).

## Results

Generally, scoliotic subjects did not differ significantly from controls and normal values. However, FVCin was below normal values (x=86.4% in 10-24° Cobb), VC and TLC means differed nonsignificantly (p=.070 and p=.074, respectively).

## Conclusion

In general, the results suggest satisfactory lung functioning, but FVCin analysis indicates inspiratory inefficiency, regardless severity of the deformation.

## Acknowledgements

This paper is a part of a research project DS.136, University School of Physical Education, Warsaw, Poland.
